# Improving multi-scale detection layers in the deep learning network for wheat spike detection based on interpretive analysis

**DOI:** 10.1186/s13007-023-01020-2

**Published:** 2023-05-13

**Authors:** Jiawei Yan, Jianqing Zhao, Yucheng Cai, Suwan Wang, Xiaolei Qiu, Xia Yao, Yongchao Tian, Yan Zhu, Weixing Cao, Xiaohu Zhang

**Affiliations:** 1 National Engineering and Technology Center for Information Agriculture, Nanjing Agricultural University, Nanjing, 210095 China; 2 Key Laboratory for Crop System Analysis and Decision Making, Ministry of Agriculture and Rural Affairs, Nanjing, 210095 China; 3 Jiangsu Key Laboratory for Information Agriculture, Nanjing, 210095 China; 4 Jiangsu Collaborative Innovation Center for Modern Crop Production, Nanjing, 210095 China

**Keywords:** Wheat spike detection, Deep learning network, Attention score, Interpretive analysis

## Abstract

**Background:**

Detecting and counting wheat spikes is essential for predicting and measuring wheat yield. However, current wheat spike detection researches often directly apply the new network structure. There are few studies that can combine the prior knowledge of wheat spike size characteristics to design a suitable wheat spike detection model. It remains unclear whether the complex detection layers of the network play their intended role.

**Results:**

This study proposes an interpretive analysis method for quantitatively evaluating the role of three-scale detection layers in a deep learning-based wheat spike detection model. The attention scores in each detection layer of the YOLOv5 network are calculated using the Gradient-weighted Class Activation Mapping (Grad-CAM) algorithm, which compares the prior labeled wheat spike bounding boxes with the attention areas of the network. By refining the multi-scale detection layers using the attention scores, a better wheat spike detection network is obtained. The experiments on the Global Wheat Head Detection (GWHD) dataset show that the large-scale detection layer performs poorly, while the medium-scale detection layer performs best among the three-scale detection layers. Consequently, the large-scale detection layer is removed, a micro-scale detection layer is added, and the feature extraction ability in the medium-scale detection layer is enhanced. The refined model increases the detection accuracy and reduces the network complexity by decreasing the network parameters.

**Conclusion:**

The proposed interpretive analysis method to evaluate the contribution of different detection layers in the wheat spike detection network and provide a correct network improvement scheme. The findings of this study will offer a useful reference for future applications of deep network refinement in this field.

## Introduction

Wheat is one of the world’s important food crops. The statistics of the Food and Agriculture Organization of the United Nations show that global wheat production in 2021 is 776.8 million tons with a planted area of 220 million hectares, and the global wheat production in 2022 is expected to be 770.8 million tons [1]. In the context of world population growth and global climate change, ensuring stable and increased wheat production is crucial to world food security. Meanwhile, because the number of wheat spikes per acre and grain weight per spike directly determine the final yield [2], detecting and counting wheat spikes are important [3] for predicting and measuring wheat yield before harvest.

With the improvement of computer technology in recent years, deep learning-based object detection techniques have been increasingly applied to wheat spike detection. Some are two-stage detection methods, e.g., R-CNN [4], Fast-RCNN [5], and Faster-RCNN [6]. Some are one-stage detection, e.g., YOLO (You only look once) [7], YOLO9000 [8], YOLOv3 [9], YOLOv4 [10], and YOLOv5 [11].

Based on these technical means, some researchers evaluated the existing methods on public datasets [12, 13], while others focused on improving the state-of-art deep-learning-based methods on their private datasets [14, 15]. In these datasets, all wheat spikes have corresponding ground-truth boxes.

Both non-convolutional and convolutional wheat spike detection methods focus on wheat spike size information. Some non-convolutional methods use image processing technology and machine learning to design feature extraction for small-sized wheat spikes detection [16, 17]. Due to the differences in variety, environment, and observation scenarios, the size of wheat spikes in images varies significantly, resulting in different roles of multi-scale detection layers of the neural network in wheat spike detection. The problem is how to quantitatively analyze the role of multi-scale detection layers in the complex network structure. Solving the problem will provide a correct direction for optimizing the wheat spike detection network, and it can also provide a reference for the research of multi-size object detection [18, 19]. The development of frontier deep learning interpretive techniques provides a reliable technical way to study this kind of problem. Selvaraju et al. proposed a method based on Gradient-weighted Class Activation Mapping (Grad-CAM), which can use the gradient information in the network back propagation along with network feature layers to generate a “visual interpretation” as the reason for decision-making of the deep learning model. It can generate the attention areas of the network layer to the specific object and use heat maps with location and semantic information to highlight the important areas in the image for predicting the conceptual object. This method provides users with explanatory results and helps them to successfully identify or optimize the stronger deep learning network [20]. In this study, we introduce a network attention method based on the Grad-CAM algorithm to explore the role of multi-scale detection layers in the deep learning model and refine the wheat detection network model according to the interpretive analysis results. First, we trained a wheat spike detection model based on YOLOv5. Then, the attention scores of each network detection layer were quantified based on the Grad-CAM algorithm. Following that, We obtained the performance of different detection layers for the detection of wheat spikes and finally clarified the optimal improvement direction of the network. In addition, the optimized wheat spike detection network was successfully constructed and validated on the Global Wheat Head Detection (GWHD) dataset.

## Methods

### Overall technical framework

**Fig. 1:**
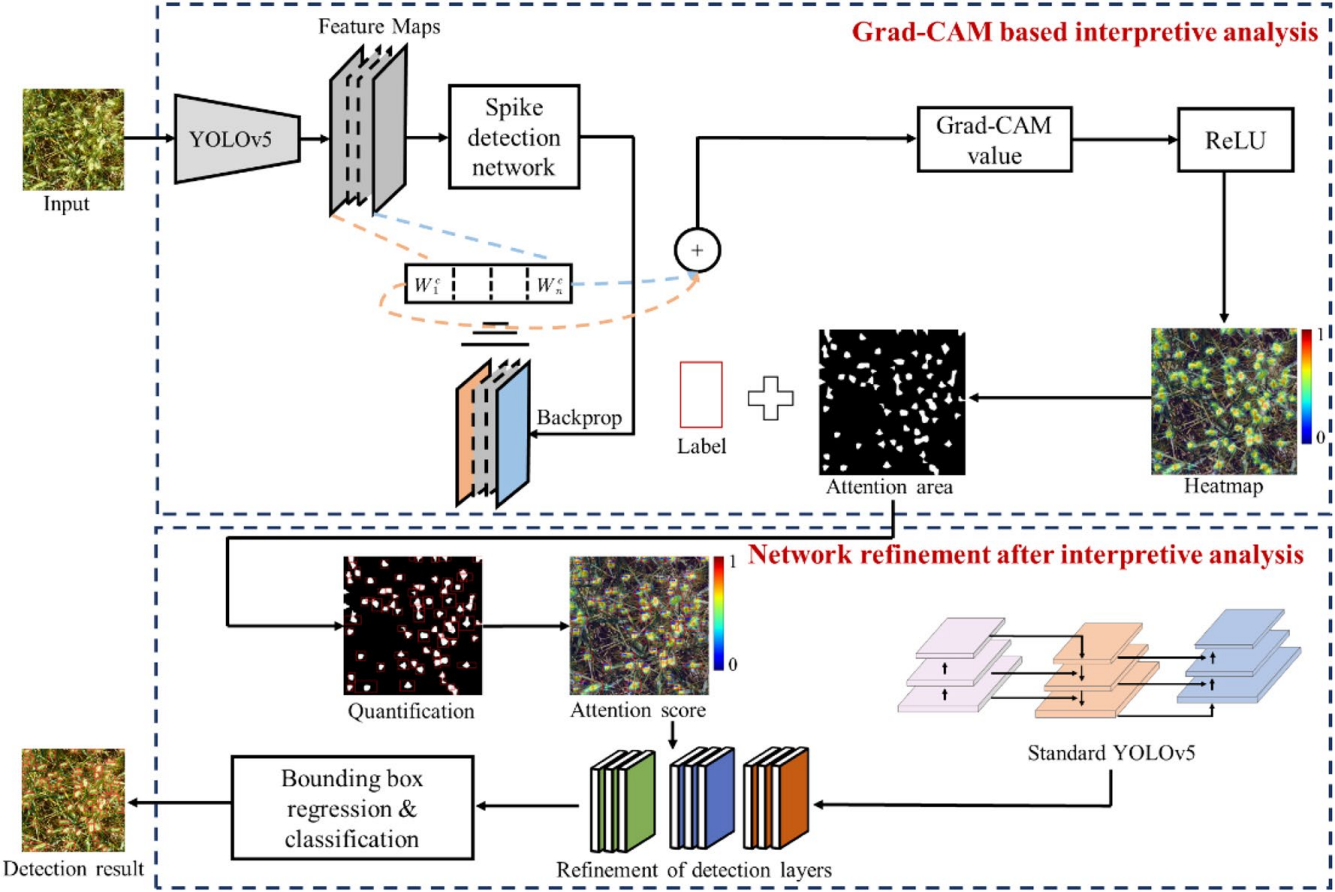
Technical framework

This study proposes a strategy for improving the detection layer scales of a deep learning-based wheat spike detection network based on interpretive analysis (Fig. 1). YOLOv5 is applied as the basic wheat spike detection network [11]. First, the feature maps of all channels in each detection layer are obtained, and the backpropagation of the detection network is performed to obtain the gradient values of each feature map to calculate the weights of feature maps. Weighted summation is conducted between the weight values and the feature map values. This process involves calculating the mean value of all pixels in an individual feature channel at each detection layer. This mean value is used as the weight parameter for the corresponding feature map. The calculated weight parameter is multiplied by the pixel values of the corresponding feature map. The results of these calculations are summed across all feature channels in the detection layer and weights of feature maps can be obtained. Second, the Grad-CAM value is input to the ReLU activation function to obtain the positive class activation mapping. Thus, the wheat spikes attention area of the network is obtained. Then, the attention score of each detection layer for an individual wheat spike is quantified by comparing the prior wheat spike labeled box and its attention area. Finally, the attention score in each detection layer is assessed to improve detection layer scales. With this improvement, a stronger wheat spike detection network is constructed to achieve higher performance in wheat spike detection.

### Global wheat head detection (GWHD) dataset

**Fig. 2:**
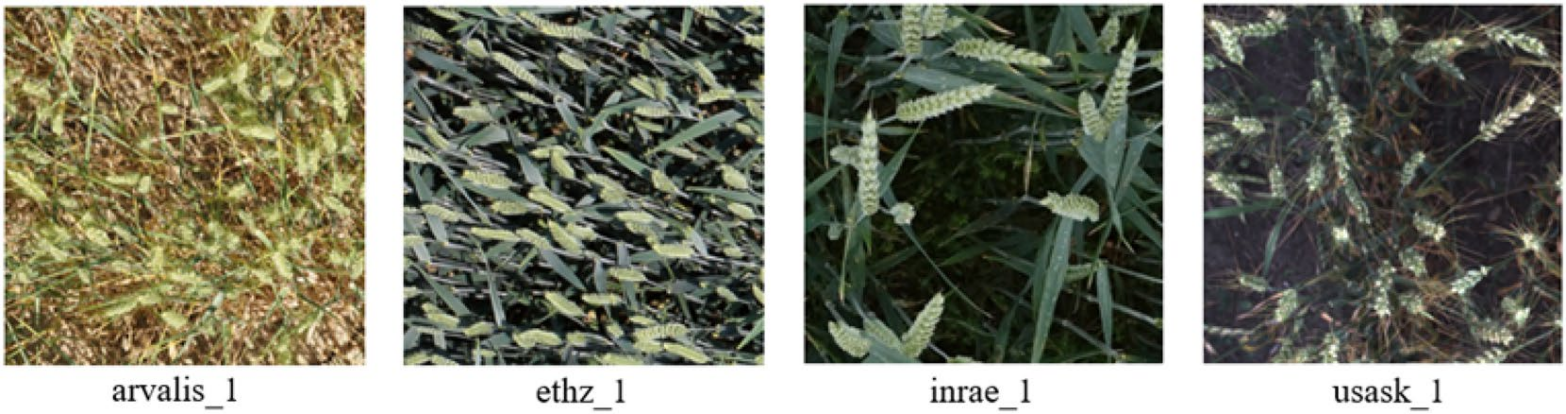
Sample wheat spike images of the four sub-datasets of GWHD_2020

GWHD dataset is an important large wheat spike image dataset in the world, covering a total of 6515 images of wheat spikes from 12 different countries, with different growth stages, genotypes, planting conditions, and image acquisition methods. The spatial resolution of images is 1024 × 1024, and the spectral bands are red, green, and blue. The total number of manually labeled wheat spikes in the dataset is 275187. The dataset consists of two versions, GWHD_2020 [21] and GWHD_2021 [22]. Among them, GWHD_2021 is an adaptation and expansion of GWHD_2020. In this study, GWHD_2021 and GWHD_2020 were both used in model development. In particular, based on the wheat spikes size distribution in the GWHD dataset, 1000 representative wheat spike images from four sub-datasets (ethz_1, arvalis_1, usask_1, and inrae_1) in GWHD_2020 were evenly selected for analyzing the role of multi-scale detection layers in the network, totaling 44538 wheat spikes. The differences in wheat spike morphology and size in the four sub-datasets are significant (Table 1, Fig. 2).

**Table 1:** The selected images from GWHD_2020 for interpretive analysis

Sub-dataset	Average spike width (Pixels)	Average spike length (Pixels)	Average spike size (Pixels)	Number of selected images	Number of labeled spikes
arvalis_1	80	75	6312	300	12,800
inrae_1	120	119	15271	176	3701
usask_1	98	87	9247	200	5737
ethz_1	76	63	4783	324	22,300
Total	–	–	–	1000	44,538

### Improvement of detection layer scale in standard YOLOv5

#### Overview of YOLOv5

The study adopts the YOLOv5 object detection model as the benchmark network. YOLOv5 is a high-performance one-stage deep learning framework. It consists of four main modules, including the input module, the backbone module, the neck module, and the detection module. The backbone module of YOLOv5 is mainly responsible for the feature extraction of wheat spikes. The neck module of YOLOv5 focuses more on image feature extraction and fusion than the backbone module. With Path Aggregation Network (PANet) and Bi-directional Feature Pyramid Network (BiFPN), the neck module achieves bottom-up and top-down feature fusion by two up-sampling operations [23, 24]. The detection module of YOLOv5 conducts object bounding box generation and object prediction in three scales: small-scale, medium-scale, and large-scale. Correspondingly, the standard YOLOv5 network contains three essential network layers: small-scale detection layer, medium-scale detection layer, and large-scale detection layer. In this study, to improve the network, we use interpretive analysis of these different scale detection layers on the performance of the wheat spike detection.

#### Interpretive analysis of detection layer scale based on Grad-CAM

We use the Grad-CAM algorithm to extract the attention areas of wheat spikes from the pre-trained wheat spike detection network on three-scale detection layers. Then, the quantitative attention scores of all wheat spikes can be obtained by comparing the prior wheat spike labeled boxes with the attention areas of wheat spikes. Finally, the contribution of each detection layer of the network to the successful detection can be quantitatively evaluated based on the attention score after calculating the proportion of wheat spikes in each score interval. Grad-CAM is a visualization interpretation method for neural networks [25]. The principle of Grad-CAM is similar to the other class activation mapping (CAM) methods. It calculates *α*_*k*_ the average value of the gradients in each channel *k* of the network feature layer as weights [26]:

1 $$a_{k} = \frac{1}{Z}\mathop \sum \limits_{i} \mathop \sum \limits_{j} \frac{{\partial y}}{{\partial A_{{ij}}^{k} }}$$

Where *y* is the prediction score of the network for the wheat spike class; $A_{ij}^{k}$ represents the value of the *i-th* row and *j-th* column in the feature map of channel *k*; *Z* represents the multiplied value of width and height of the feature map.

Then, the weighted summation operation of weight *α*_*k*_ and feature map *A*_*k*_ is performed on these channels, and the ReLU activation function filters out the negative values of the feature layer to obtain the final Grad-CAM value *L*_*Grad−CAM*_:

2 $$L_{{Grad - CAM}} = {\text{ReLU}}\left( {\mathop \sum \limits_{k} \alpha _{k} A^{k} } \right)$$

where *A*_*k*_ represents the *k-th* channel in the feature layer *A*; *α*_*k*_ represents the weight of the *k-th* channel in the feature layer.

Grad-CAM values are visualized in heat maps, thus visualizing the role of important areas in the network on wheat spike detection. Meanwhile, the Grad-CAM value is applied to extract the attention area to wheat spikes of three-scale detection layers. We define the attention area *R*_*Grad−CAM*_ as the region with a non-empty Grad-CAM value area and is quantitatively compared with the area *R*_*Label*_ f prior wheat spike labeled boxes. Then the *S* value is derived as the contribution of detection layers to the wheat spike detection:

3 $$S=\frac{{R}_{Grad-CAM}}{{R}_{Label}}$$

where *R*_*Grad−CAM*_ represents the area of attention region; *R*_*Label*_ epresents the area of wheat spike labeled boxes; *S* represents the final attention score.

#### Network improvements

**Fig. 3:**
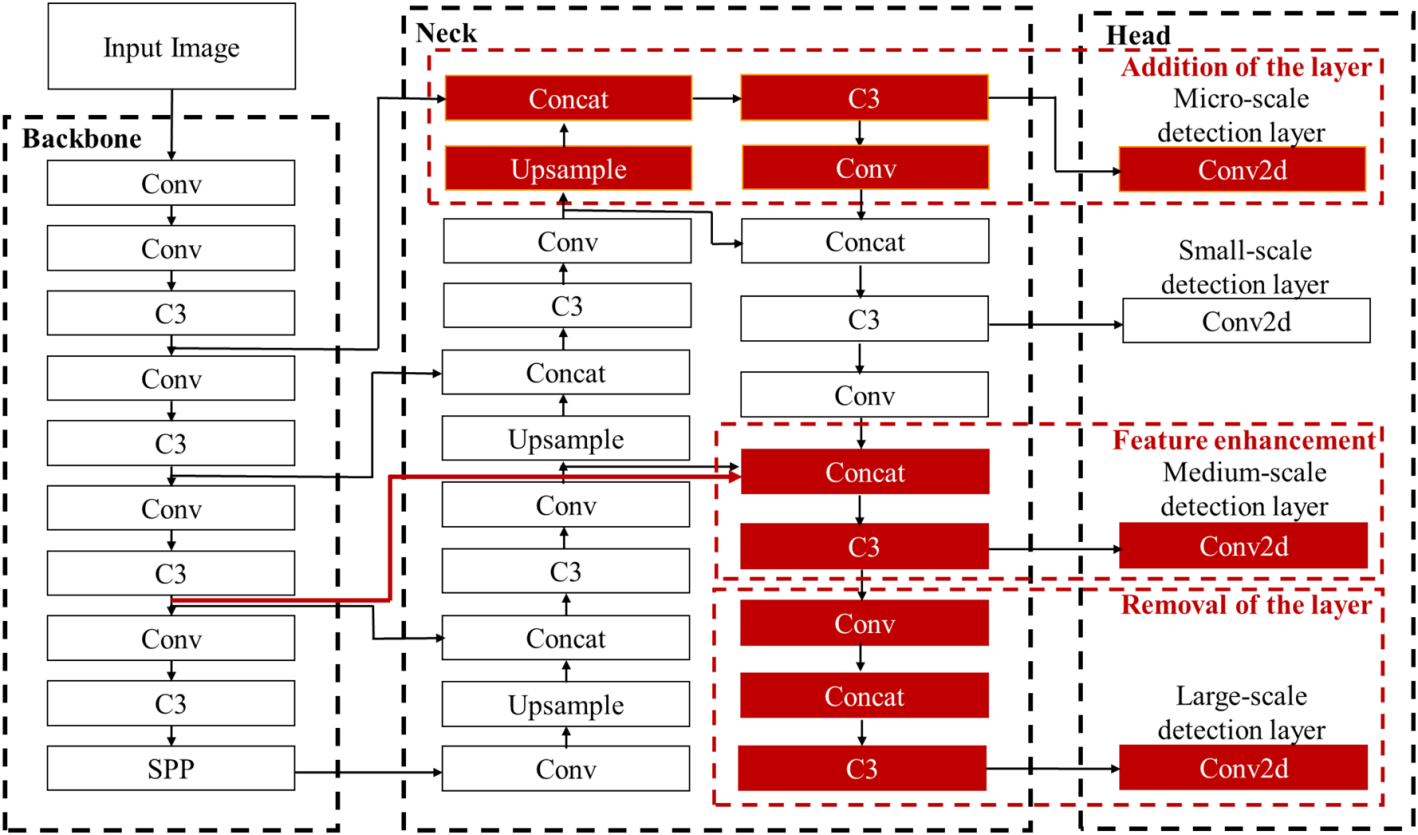
Measures of improving the detection layer scale for wheat spike detection: white parts represent standard YOLOv5, and red parts represent network improvements

The proposed method quantitatively evaluates the performance and contribution of the original three detection layers based on the attention scores. Based on this evaluation, we develop a network improvement strategy. Removal of the large-scale detection layer will be considered when its attention score is poor. Adding a micro-scale detection layer will be considered to improve the detection of small-sized objects when the attention score of the small-scale detection layer is excellent [27]; otherwise, it is removed. When the attention score of the medium-scale detection layer is excellent, the feature enhancement operation will be applied. In the Yolov5 backbone, shallow convolutional layers can extract spatial features, while deep convolutional layers can extract semantic features. The semantic and spatial features are upsampled and downsampled, respectively, and combined bidirectionally by fusion. The multi-scale features are then directed to the medium-scale detection layer. This process introduces more feature information with the same scale from the backbone module to the neck module and enhances the features in the medium-scale detection layer. These measures build a new strong detection network for wheat spike objects (Fig. 3).

### Experimental settings

#### Multi-resolution training strategy

The study adopts a multi-resolution training strategy. The network is trained by inputting images with different resolutions of 150 × 150, 300 × 300, 450 × 450 and 600 × 600 to obtain the trained model.

The experiment is conducted on a workstation equipped with Intel^®^ i7 10,700 processor, NVIDIA^®^ Geforce GTX 1080Ti graphics processor (12GB memory), 32GB RAM, and 1TB storage. The computer operating system is Ubuntu 16.06, and the hyperparameter settings for network training are set as follows (Table 2). Batch size, training round, learning rate, and momentum are separately set to 8, 100, 0.01, and 0.9.

**Table 2:** Network training hyperparameter setting

Input size	Batch size	Epoch	Learning rate	Momentum
150 × 150	8	100	0.01	0.9
300 × 300	8	100	0.01	0.9
450 × 450	8	100	0.01	0.9
600 × 600	8	100	0.01	0.9

#### Evaluation metrics

The study adopts *precision*, *recall,* and average precision (*AP*) to evaluate the performance of the deep-learning network model for wheat spike detection. The *precision* and *recall* are defined as:

4 $$precision=\frac{TP}{FP+TP}$$

5 $$recall=\frac{TP}{FN+TP}$$

where *TP*, true positive, means that positive samples are correctly predicted as positive; *FP*, false positive, means that negative samples are incorrectly predicted as positive; *FN*, false negative, means that positive samples are incorrectly predicted as negative.

Since *precision* and *recall* are a pair of indicators that affect each other, it is difficult to fully evaluate the network using one of the two indicators alone. Therefore, the average precision (*AP*) is introduced. *AP* is the average *precision* of *recall* in the 0–1 interval for detecting a certain class of objects and obtained by:

6 $$AP={\int }_{0}^{1}precision(recall)drecall$$

## Results

### Attention scores of each detection layer

**Fig. 4:**
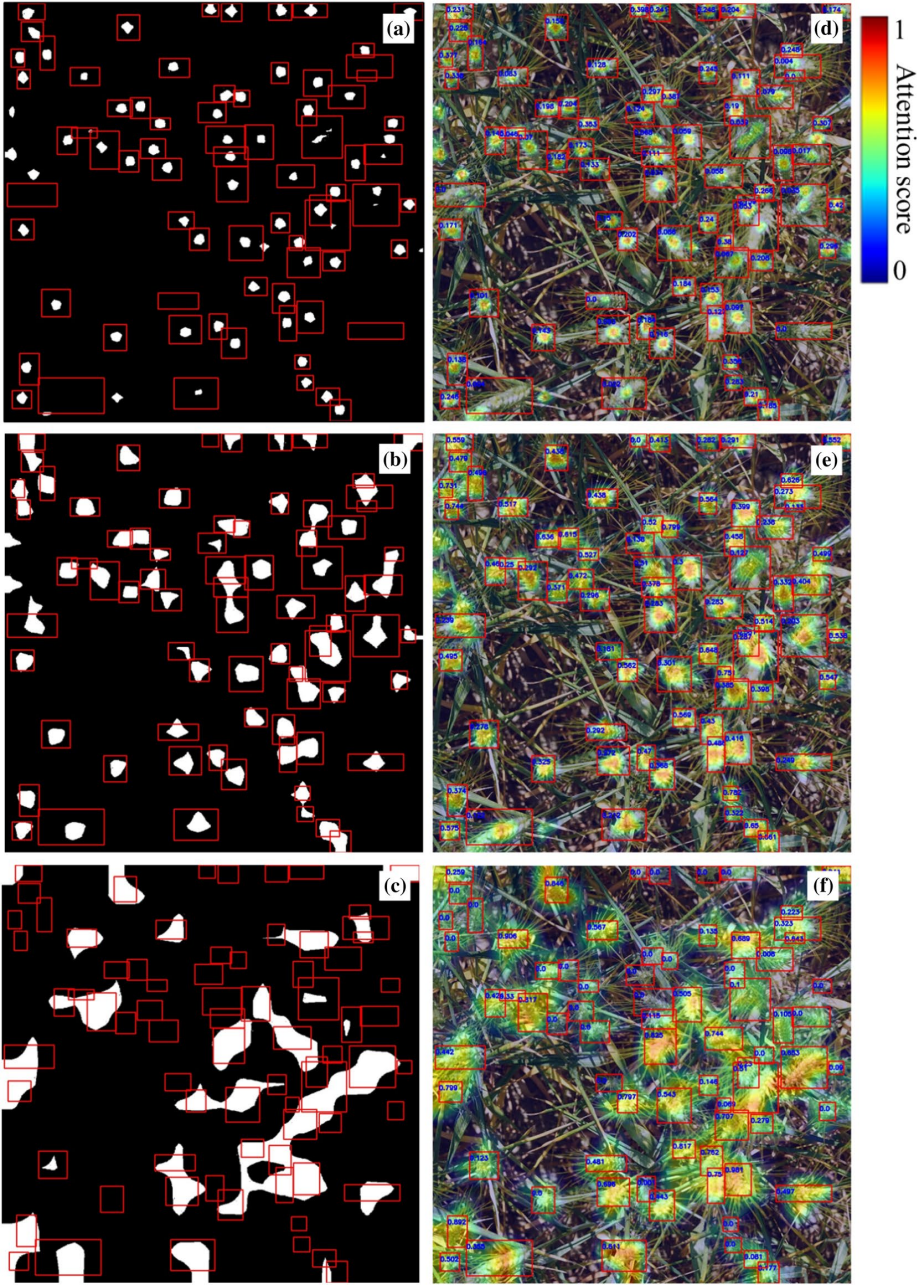
Attention areas and attention scores of multi-scale detection layers (red rectangles are the prior labeled wheat spike bounding boxes). **a** Attention areas of the small-scale detection layer (in white). **b** Attention areas of the medium-scale detection layer (in white). **c** Attention areas of the large-scale detection layer (in white). **d** Attention score values and heatmaps of the small-scale detection layer. **e** Attention score values and heatmaps of the medium-scale detection layer. **f** Attention score values and heatmaps of the large-scale detection layer

Experimental results show that attention areas of wheat spikes in the small-scale detection layer were small, and therefore the calculated attention scores are relatively low (Fig. 4a, d).

**Fig. 5:**
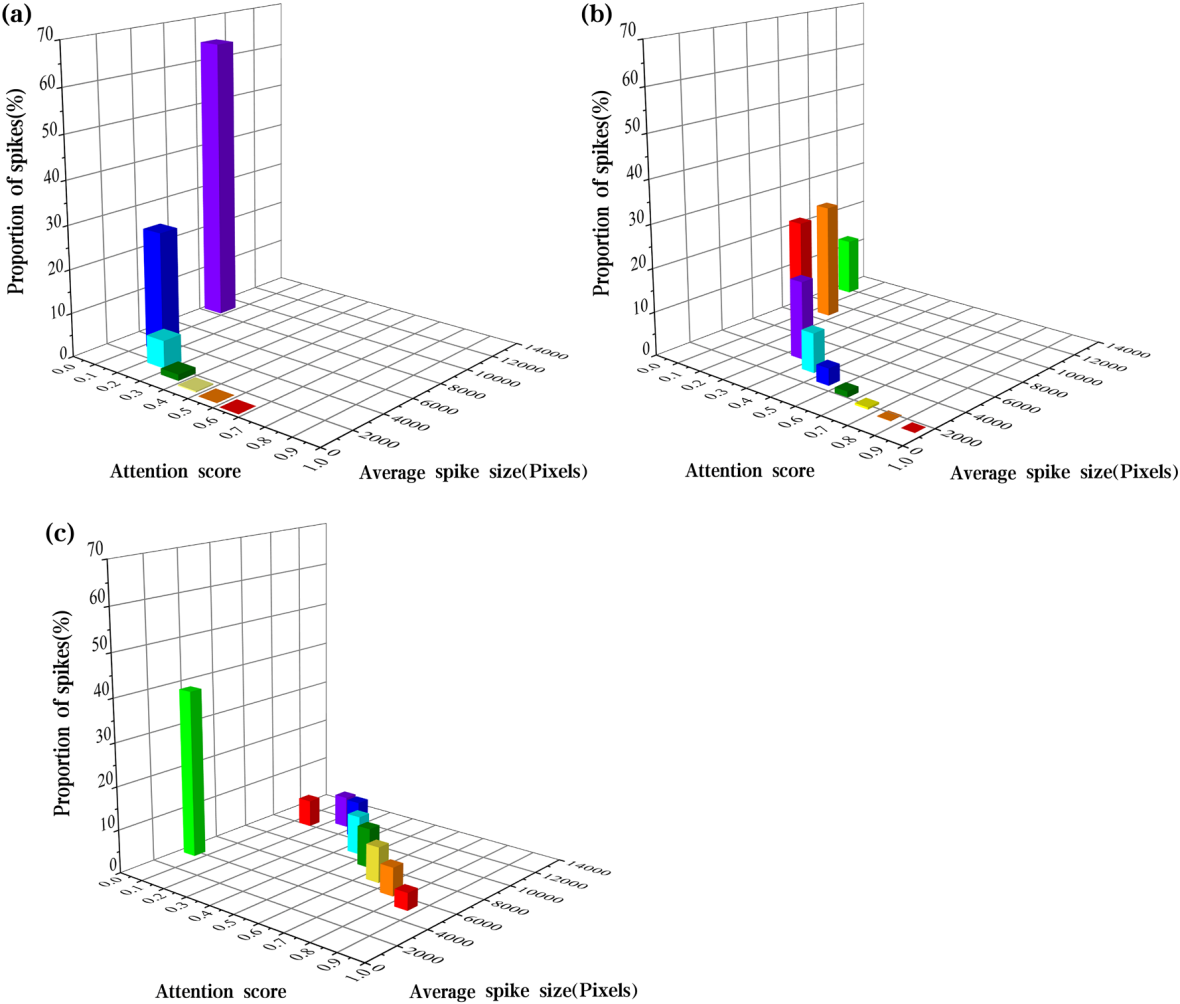
Spike distribution in different attention score ranges of three-scale detection layers. **a** Small-scale detection layer. **b** Medium-scale detection layer. **c** Large-scale detection layer

Moreover, the statistics of attention scores of the small-scale detection layer show a trend of higher scores for smaller sizes and weaker attention for larger wheat spikes (Table 3, Fig. 5). Most wheat spikes have low attention scores in the range of 0.0–0.1 and 0.1–0.2 (64.4% and 27.5%). They have 8682 and 3438 pixels in size. The proportion of wheat spikes in the 0.2–0.7 score interval is small, while these wheat spikes are also small, with a size below 2137 pixels. Moreover, there are no wheat spikes in the score interval 0.8–1.0.

**Table 3:** Attention score statistics of multi-scale detection layers

Detection layer	Attention score	Proportion of spikes (%)	Average spike size (Pixels)	Total attention score range	Mean attention score
Small-scale detection layer	0.0–0.1	64.4	8682	0–0.607	0.083
	0.1–0.2	27.5	3438		
	0.2–0.3	6.3	2137		
	0.3–0.4	1.4	1581		
	0.4–0.5	0.3	1188		
	0.5–0.6	0.07	961		
	0.6–0.7	0.03	733		
	0.7–0.8	–	–		
	0.8–0.9	–	–		
	0.9–1.0	–	–		
Medium-scale detection layer	0.0–0.1	13.0	12030	0–0.984	0.25
	0.1–0.2	26.4	8949		
	0.2–0.3	27.9	5619		
	0.3–0.4	17.8	4165		
	0.4–0.5	9.0	3360		
	0.5–0.6	3.9	2758		
	0.6–0.7	1.4	2347		
	0.7–0.8	0.5	2003		
	0.8–0.9	0.08	1742		
	0.9–1.0	0.02	1527		
Large-scale detection layer	0.0–0.1	38.8	3891	0–1	0.309
	0.1–0.2	6.3	10298		
	0.2–0.3	7.3	11365		
	0.3–0.4	8.7	10581		
	0.4–0.5	9.0	8946		
	0.5–0.6	9.2	7962		
	0.6–0.7	8.2	6861		
	0.7–0.8	6.6	6092		
	0.8–0.9	4.0	5297		
	0.9–1.0	1.9	4346

In Fig. 4, 75 wheat spikes are labeled, and attention areas of wheat spikes are visually larger and more accurate in the medium-scale detection layer than in the small-scale detection layer (Fig. 4b, e). Attention scores of the medium-scale detection layer are more evenly distributed and show a trend of higher scores for smaller wheat spikes (Table 3, Fig. 5). Moreover, most wheat spikes have a moderate score in the range of 0.1 to 0.4. These wheat spikes also have a medium size. The proportion of wheat spikes in the 0.4–1.0 interval is small, and the sizes of wheat spikes in this interval are smaller, below 3360 pixels.

**Fig. 6:**
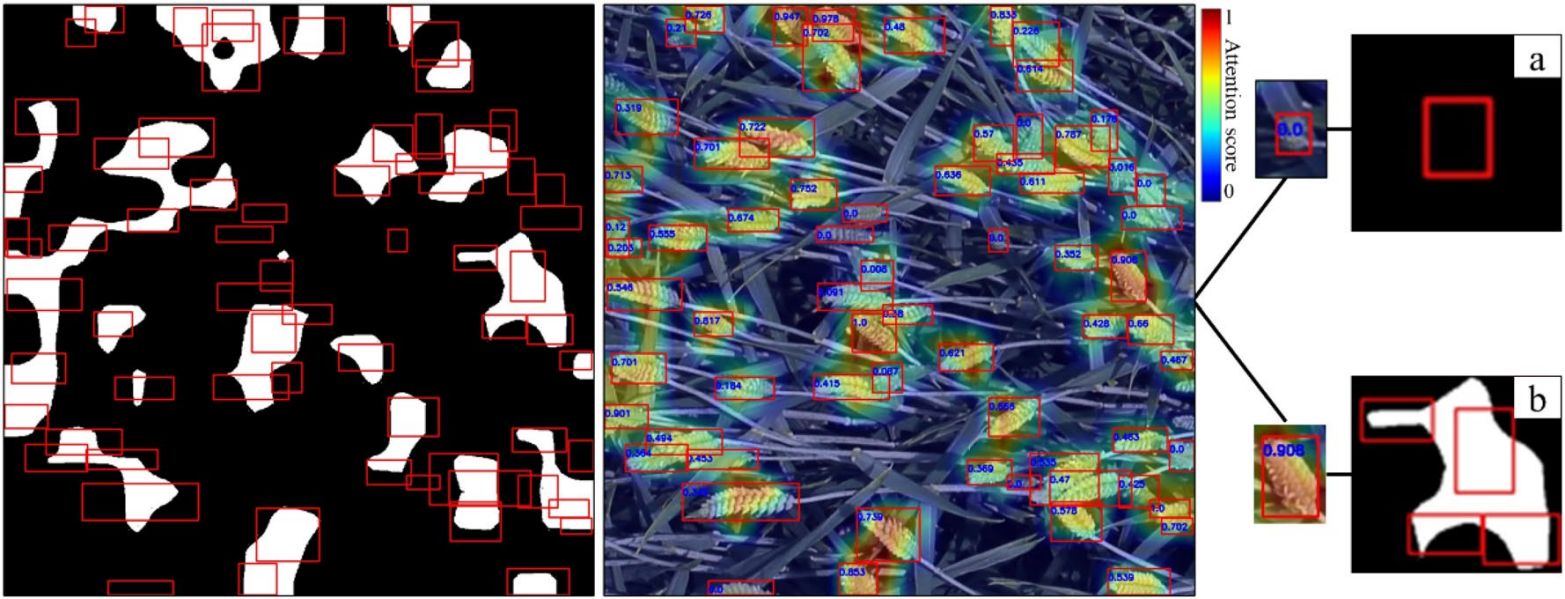
Two typical problems in the large-scale detection layer. **a** Absence of network attention for a small-sized wheat spike in this detection layer. **b** The attention area of this detection layer significantly confuses the wheat spike with the non-spike background region. Regions of white/black pixels are network attention areas and backgrounds separately.

Attention scores of the large-scale detection layer indicate that this layer weakens wheat spike detection (Fig. 4c, f). The largest proportion of wheat spikes is in score interval 0.0–0.1, with a proportion of 38.8%. Besides, these spikes are small, with an average size of 3891 pixels. In the remaining score intervals, wheat spikes are evenly distributed (Table 3, Fig. 5). Attention areas of wheat spikes in the large-scale detection layer are visually large. There are many wheat spike labeled boxes without existing attention areas. In addition, there is a phenomenon that attention areas exceeded labeled boxes. It indicates that the network confuses background areas with wheat spike areas. Therefore, the network cannot make accurate inferences (Fig. 6).

Wheat spikes with an attention score of 0 in three detection layers are counted (Table 4). The three detection layers have 17.5%, 4.0%, and 30.0% wheat spikes with an attention score of 0. It indicates that the deep network failed to focus on this part of areas where existing wheat spikes. Three parts of wheat spikes have average sizes of 13040, 4660, and 3434 pixels. The small-scale detection layer has difficulty identifying larger wheat spikes, while the large-scale detection layer has difficulty identifying smaller ones. Medium-scale detection layer merely ignores 4% of all wheat spikes. It achieves the best performance among the three detection layers. The small-scale detection layer also performs better for small-sized wheat spikes than for large ones.

**Table 4:** Spikes with an attention score of 0 in three detection layers

Detection layer	Mean spike size (Pixels)	Proportion of spikes (%)
Small-scale	13040	17.5
Medium-scale	4660	4.0
Large-scale	3434	30.0

Particularly, the large-scale detection layer ignores 30% of small-sized wheat spikes with an average size of 3434 pixels and misclassifies background areas as wheat spikes. It has difficulty distinguishing spike/background areas in Fig. 6 where 67 wheat spikes are labeled.

### The performance of the improved network

In summary, the large-scale detection layer performs the worst, while the medium-scale and small-scale detection layers are relatively better. Therefore, the network structure is streamlined by removing the large-scale detection layer, enhancing feature fusion in the medium-scale detection layer, and adding a micro-scale detection layer to enhance the network’s performance in detecting small-sized wheat spikes.

**Fig. 7:**
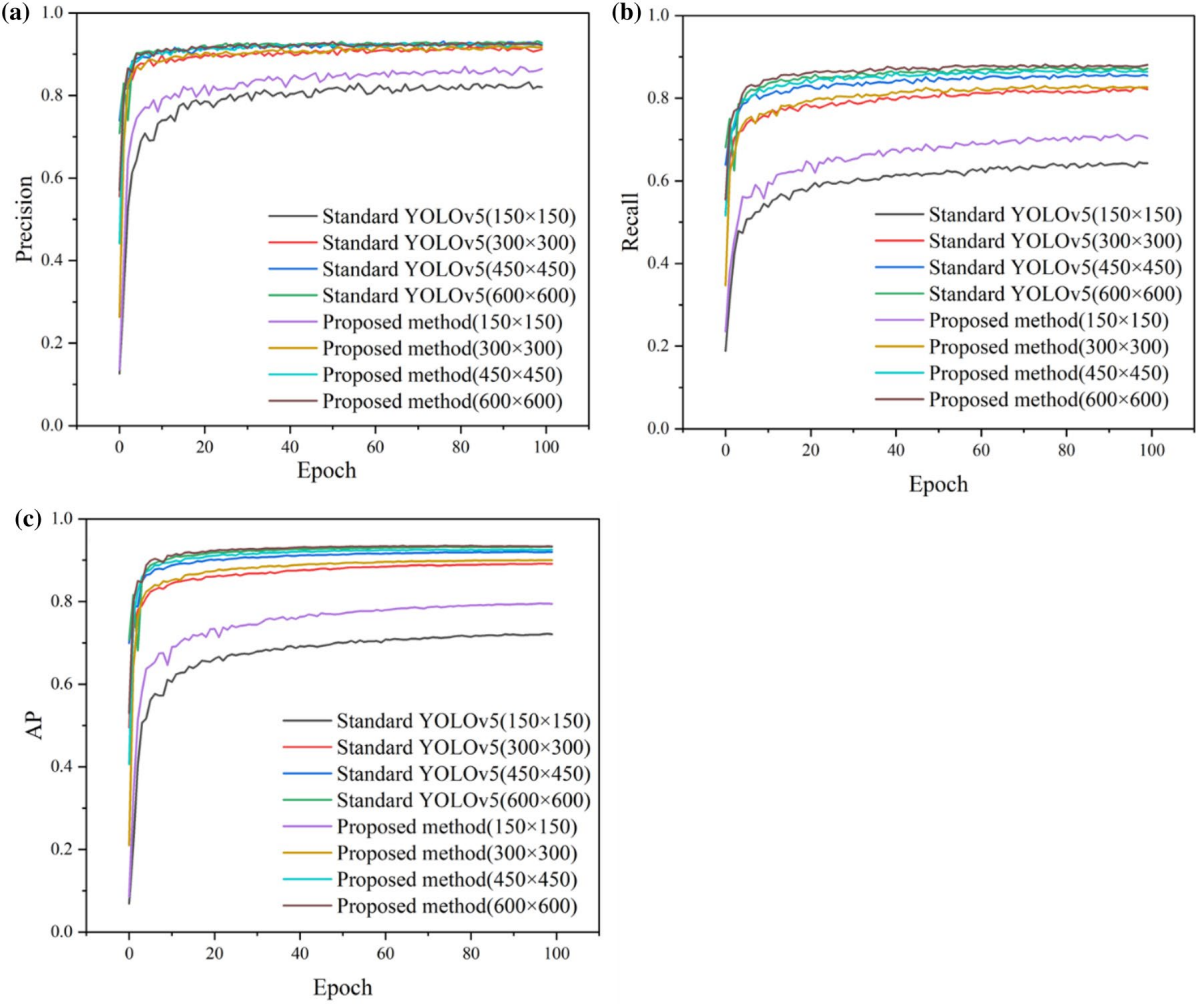
*Precision*, *Recall*, and *AP* curves of the wheat spike detection for the improved method and standard YOLOv5. **a** The precision curves. **b** The recall curves. **c** The *AP* curves

We compare the standard YOLOv5 and the improved network on the GWHD dataset (Fig. 7). The improved network increases AP by 0.5% compared to standard YOLOv5 and achieves the best AP of 93.5% on the 600 × 600 resolution image. The largest improvement is achieved in the 150 × 150 resolution image training, with an AP improvement of 7.4%. The wheat spike detection network can be improved based on the proposed interpretive analysis. Furthermore, although the proposed method results in a slight decrease in FPS to 130 and a slight increase in the number of network layers to 236, the network parameters are reduced from 7 to 6 M, and AP is improved in each input size (Table 5).

**Table 5:** The performance of the improved network vs. standard YOLOv5

Method	Input size	FPS	Layers	Parameters	AP (%)
Standard YOLOv5	150 × 150	166	213	7 M	72.2
	300 × 300	166	213	7 M	89.2
	450 × 450	166	213	7 M	92.0
	600 × 600	166	213	7 M	93.0
Improved network	150 × 150	130	236	6 M	79.6
	300 × 300	130	236	6 M	90.0
	450 × 450	130	236	6 M	92.5
	600 × 600	130	236	6 M	93.5

## Discussion

Scale issue has always been an important research problem in wheat spike detection, similar to other object detection tasks [28, 29]. The scale issue in the wheat spike detection network exists in terms of the multi-scale input images and the multi-scale of network layers. They cause an impact on the construction of the wheat spike detection network, including the model efficiency and performance. Therefore, it is necessary to carry out interpretive analysis and scale optimization of the network. Due to the limitations of different image acquisition platforms, there is an established problem that objects vary in size due to their physical morphology in covering datasets. The size of wheat spikes in images varies significantly in the study of wheat spike detection. Some researchers directly start from the size characteristics of objects in datasets and determine the optimal pixel size by up-sampling the small objects in original images. It effectively improves detection accuracy, but too many upsampling operations increase the processing time and lead to more false detections [30]. Based on the above, relevant studies deliberately select labeled datasets with significant differences in object scales [31] and sufficient data amount [32, 33] in the preliminary dataset preparation stage for detection. The proposed Feature Pyramid Network (FPN) solves the multi-scale object detection problem at the network structure level. The problem is successfully settled by building an FPN structure for multi-scale detection [34].

Adjusting network structure will affect object detection accuracy for a deep learning network [35]. Based on subjective experience, researchers have enhanced the detection network’s performance by adding a micro-scale detection layer [36], adjusting feature enhancement modules [37–43], and rotating original horizontal detection boxes [44–46]. However, the studies mentioned above focused merely on the direct application of prior knowledge and thus lacked significant support from interpretive works [47].

**Fig. 8:**
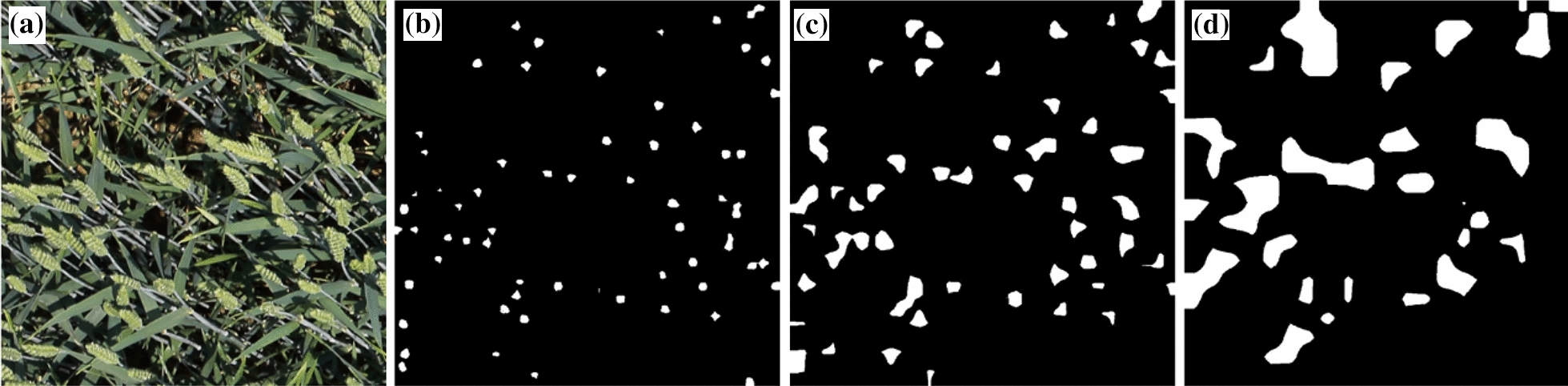
Attention area of different scale detection layers (in white): **a** Original wheat spike image. **b** Attention area of the small-scale detection layer. **c** Attention area of the medium-scale detection layer. **d** Attention area of the large-scale detection layer

Most interpretive research provides qualitative explanations by outputting saliency maps of a network to provide a sound scientific basis for network refinement [48–50]. With saliency maps, researchers can visualize the location and size of network attention areas [51]. However, quantitative metrics are lacking in these studies for further network performance evaluation. In the proposed research, attention areas extracted from different scale detection layers show significant scale effects. They can accurately reflect semantic and location information of wheat spikes in each detection layer (Fig. 8). With the Grad-CAM algorithm, we successfully quantitatively describe the scale effects and provides a scientific basis for the scale optimization of the network.

It is visually evident that the attention area of small-scale and medium-scale detection layers accurately reflects wheat spikes’ morphology and spatial location. Small-scale and medium-scale detection layers successfully focus on 82.5% and 96% of wheat spike objects in all 44538 wheat spikes. Attention areas of the large-scale detection layer are far beyond areas where wheat spikes locate. The large-scale detection layer focuses on merely 30% of wheat spikes, and there is confusion between wheat spike areas and background areas (Fig. 8). This confusion means that the wrong attention is paid to non-spike areas. It may be related to the receptive field of the neural network. The size in pixels of feature maps output by each detection layer decreases exponentially with a factor of 4 from the small-scale to the large-scale detection layer. Meanwhile, the corresponding size in pixels of receptive fields increases exponentially with a factor of 4 [52, 53]. It is consistent with the situation presented in the graph (Fig. 8).

Moreover, according to interpretive analysis, the large-scale detection layer performs poorly in detecting wheat spikes in the GWHD dataset. The new network without a large-scale detection layer achieves overall improvements in all result metrics in multi-resolution jobs (Table 6). This finding is consistent with other research results achieving better results in higher resolution training [54].

**Table 6:** The performance of the standard YOLOv5 vs. YOLOv5^a^

Method	Input size	FPS	Layers	Parameters	AP (%)
YOLOv5	150 × 150	166	213	7 M	72.2
	300 × 300	166	213	7 M	89.2
	450 × 450	166	213	7 M	92.0
	600 × 600	166	213	7 M	93.0
YOLOv5^a^	150 × 150	188	190	5 M	74.9
	300 × 300	188	190	5 M	89.4
	450 × 450	188	190	5 M	92.5
	600 × 600	188	190	5 M	93.3

^a^YOLOv5^a^ represents the standard YOLOv5 without a large-scale detection layer

This study aims to explore the combination of wheat spike features and interpretability methods to construct a wheat spike detection network. This is a general improvement method that can be applied to various single-stage object detection models, including YOLOv5, YOLOv6 and the latest YOLOv7. Existing object detection models are evolving towards large-scale and universal models with massive parameters, making training difficult and leading to high computational costs [55, 56]. This paper integrates interpretable methods to construct and optimize a wheat spike detection model for complex scenes without too many parameters, providing theoretical foundations for model development (Table 5).

The study has only carried out interpretive research on three-scale detection layers and conducted scale refinement for these layers. In future work, it will be meaningful to introduce attention-based interpretive work on the network’s backbone module to explore its improvement path. We also plan to further explain how the convolutional layers and kernels in the neural network affect the accuracy of wheat spike detection. Meanwhile, more diverse wheat spike datasets are needed to validate our method to ensure a convincing and objective research finding.

## Conclusion

The study proposes a scale refinement method for the detection layers of the wheat spike detection network based on the deep learning interpretive method Grad-CAM. A more streamlined wheat spike detection network is successfully constructed and performs well on the GWHD dataset with better detection accuracy and lower model complexity. Compared to previous work, our study has two novel aspects. First, the proposed method integrates features with prior knowledge without directly referencing and superimposing novel technologies in object detection. By analyzing the size features of wheat spikes, we design a superior wheat spike detection network. Second, we demonstrate the effectiveness of the improved modules from both theoretical and experimental perspectives. The size characteristics of wheat spikes in the dataset are quantitatively analyzed and the results are used to optimize the wheat spike detection network. The study provides a new theoretical basis for research on wheat spike detection based on deep learning. It offers a technical reference for constructing and developing wheat spike detection networks with better robustness, generality, and applicability.

## Data Availability

The datasets generated and/or analyzed during the current study are available from the corresponding author upon reasonable request.
